# Temporal Progression of Pneumonic Plague in Blood of Nonhuman Primate: A Transcriptomic Analysis

**DOI:** 10.1371/journal.pone.0151788

**Published:** 2016-03-22

**Authors:** Rasha Hammamieh, Seid Muhie, Richard Borschel, Aarti Gautam, Stacy-Ann Miller, Nabarun Chakraborty, Marti Jett

**Affiliations:** 1 US Army Center for Environmental Health Research, Fort Detrick, Maryland, 21702–5010, United States of America; 2 Advanced Biomedical Center, Fredrick National Lab for Cancer Research, National Cancer Institute, Fredrick, Maryland, 21702, United States of America; 3 Walter Reed Army Medical Center, Silver Spring, Maryland, 20910, United States of America; 4 The Geneva Foundation, Fort Detrick, Maryland, 21702–5010, United States of America; CEA, FRANCE

## Abstract

Early identification of impending illness during widespread exposure to a pathogenic agent offers a potential means to initiate treatment during a timeframe when it would be most likely to be effective and has the potential to identify novel therapeutic strategies. The latter could be critical, especially as antibiotic resistance is becoming widespread. In order to examine pre-symptomatic illness, African green monkeys were challenged intranasally with aerosolized *Yersinia pestis* strain CO92 and blood samples were collected in short intervals from 45 m till 42 h post-exposure. Presenting one of the first genomic investigations of a NHP model challenged by pneumonic plague, whole genome analysis was annotated *in silico* and validated by qPCR assay. Transcriptomic profiles of blood showed early perturbation with the number of differentially expressed genes increasing until 24 h. By then, *Y*. *pestis* had paralyzed the host defense, as suggested by the functional analyses. Early activation of the apoptotic networks possibly facilitated the pathogen to overwhelm the defense mechanisms, despite the activation of the pro-inflammatory mechanism, toll-like receptors and microtubules at the port-of-entry. The overexpressed transcripts encoding an early pro-inflammatory response particularly manifested in active lymphocytes and ubiquitin networks were a potential deviation from the rodent models, which needs further verification. In summary, the present study recognized a pattern of *Y*. *pestis* pathogenesis potentially more applicable to the human system. Independent validation using the complementary omics approach with comprehensive evaluation of the organs, such as lungs which showed early bacterial infection, is essential.

## Introduction

Extracellular proliferation and rapid propagation of *Yersinia* distinguishes its pathogenesis from other enteropathogenic agents, such as *Salmonella* and *Shigella* [1–3]. The capability of *Yersinia pestis (Y*. *pestis)* to rapidly multiply, coupled with its atypical symptoms at the initial stage of infection, presents daunting challenges for initiating immediate therapeutic action and appropriate preventative care. *Y*. *pestis*, the causative agent of a pneumonic plague, manifests early flu-like symptoms that evolve into chest pain, shortness of breath, cough, and bloody or watery sputum. If untreated within the first 20 hours of onset of symptoms, it is almost always fatal due to respiratory failure and/or circulatory collapse, coagulopathy, and hemorrhage [4]. Its alarmingly high mortality rate, even under antimicrobial treatment, and its easy transmission and aerosol characteristics continually consider *Y*. *pestis* as a major threat to public health [5] and potential agent of bioweapon [6]. Recent fatality caused by *Y*. *pestis* infection has been attributed to the clinical misdiagnosis and misidentification by multiple automated platforms further underlines the need for a reliable early detection technique [7].

*Y*. *pestis* is a rod-shaped Gram-negative intracellular bacterium that, once inhaled, first encounters phagocytosis mediated by macrophages and neutrophils [2,8]. Surviving pathogens inside the macrophages proliferate, continually expressing various virulence factors such as Yops, the F1 antigen, and the V antigen. Once released into the blood stream, the extracellular pathogens can rapidly gain access to many organs and systematically modulate the innate and adaptive immune responses. Pathological analysis reported a rapid dissemination of *Y*. *pestis* in murine lungs within 1 h after the intranasal challenge that developed into a splenic infection within 36 h [9]. An *ex vivo* bioluminescence study found *Y*. *pestis* proliferated in lungs 1 d after intranasal challenge; within 24 h, pathogens had spread to the spleen and liver, and by 72 h the entire body was infected [10]. Interestingly, no viable pathogens were detected in rodent [11] and primate [12] blood until 72 h post-exposure.

A comprehensive investigation of the early host-pathogen dynamics would be of a great importance, particularly in regard to biological warfare and clinical profiles. The study of the infection trajectory focused on the important clinical and molecular landmarks of disease progression could be highly insightful, yet is largely lacking. The *in vivo* chain of events following the inhalation of *Y*. *pestis* is still poorly understood, especially in the case of large mammals, thereby presenting a critical knowledge gap [13,14].

In the past, omics approaches were used to interrogate the longitudinal murine pneumonic plague model [15,16], while increasing interest has been shown on its early pathogenesis [17–19]. Similar to the distinguishing signature of the bubonic plague model [20,21], pneumonic plague in rodents manifested a biphasic mode of temporal progression in lungs, kidney and spleen tissues [9,22]. Here, the early pre-inflammatory phase was found to have reduced transcriptomic recruitment until 12 h post infection, followed by a pro-inflammatory augmentation that resulted in a larger number of genes dysregulated after 48 h post-challenge [15]. The majority of the inflammation markers was overexpressed in blood, lungs, spleen, liver and heart at 72 h post-infection [11,15]. A similar observation was also reported in rats challenged intranasally with *Y*. *pestis* [23].

Investigation of the nonhuman primates that share phylogenetic proximity to humans becomes essential to bridge the lack of clinical knowledge about *Y*. *pestis*. In the present study, we characterized the longitudinal progression of pneumonic plague in African green monkeys (*Chlorocebus aethiops*) exposed to *Y*. *pestis* strain CO92 via inhalation challenge. Blood draws were designed with shorter intervals, starting at 45 m post-exposure, to take a more detailed look at the disease trajectory. Layton et al., [12] characterized this animal model as a viable substitute for human testing according to the FDA ‘Animal Rule’. They observed that the bacterial load became detectable in blood in a little less than three days. On average, the animals survived another day or two. By then, the pathogen was widely disseminated in tissues such as the lymph nodes, lungs, liver, and spleen [12]. Following the published animal handling guidelines [12,24], we collected blood at eight time points from the moribund animals, starting from 45 m until 42 h. High throughput transcriptomic outcome was annotated *in silico* and validated by qPCR assay.

## Materials and Methods

### Ethics Statement

All animal experiments were approved by the Institutional Animal Care and Use Committee (IACUC) at the Walter Reed Army Institute of Research (WRAIR), Silver Spring, MD, and were performed in a facility accredited by the Association for the Assessment and Accreditation of Laboratory Animal Care International (AAALAC). The approved protocol was PO01-08: Systems biology studies to identify host indicators /therapeutic targets at early time periods post exposure to Y. *pestis* (CO92) in African Green Monkeys (*Chlorocebus aethiops*)

### Animals

The animals were obtained from the primate colony of WRAIR. Adult male African green monkeys (*Chlorocebus aethiops*) of 4.8–7.0 kg body weight were TB-negative, SIV-negative, and SRV-negative with serum titers of anti-*Yersinia* IgG antibodies <1:160. Only monkeys in good health as shown by physical examination, complete blood counts, and serum chemistry profiles were used. Monkeys were individually housed in 3 × 2.6 × 2-ft. squeeze cages in free-standing enclosures equipped with standard enrichments, exposed to ambient environmental conditions inside an Animal Biosafety Level 3 (ABSL-3) containment laboratory, which approximated a 12:12-hour light/dark cycle with temperatures between 25°C and 30°C. Monkeys were fed a standard laboratory primate chow (TekLad, Madison, WI) with water provided *ad libitum* by Lixit valve (Lixit Corp., Napa, CA). Veterinary care was provided twice a day, but not more than 12 h interval to the healthy animals. Once exposed, the interval time was reduced to not longer than 2 h.

### Overall experimental design (S1 Fig)

Following a published report [12], the present study was designed to expose the nonhuman primates with a target dose of 100 ± 50 LD_50_ aerosolized *Y*. *pestis* strain CO92 under ABSL-3 conditions. Seventy-two hours prior to aerosol exposure, all animals were moved into the ABSL-3 for acclimatization. As described in S1 Fig, initial blood draws were carried out 24 h before *Y*. *pestis* aerosol challenge. The animals fasted for last 6 h and were anesthetized using 4 mg/kg Telazol (Fort Dodge Animal Health, Fort Dodge, IA); after 15 m, they were aerosol challenged. Post-exposure clinical illness and disseminated infection were evaluated by direct clinical observations. All animals within each group were exposed on the same day at 30-minute intervals, and subsequent blood draws were conducted at 45 m, 6 h, 9 h, 12 h, 18 h, 24 h, 32 h and 42 h post-exposure.

Except for the 45 m blood draw, the remaining blood draws were terminal with the animals immediately euthanized to collect organs. For the 45 m and 6 h blood draws, we used the same cohort. The blood draws were carried out at the same time on the scheduled days to minimize molecular variances attributed to the circadian cycle. All animals were humanely euthanized by first inducing a deep plane of anesthesia with intramuscular administration of 9 mg/kg Telazol. Euthasol (390 mg/ml pentobarbital and 50 mg/ml phenytoin (Virbac Corp., Fort Worth, TX, IC)) was administered at a dose of 60 mg/kg pentobarbital sodium using an 18-gauge needle. The animal was auscultated to determine the absence of heartbeat and breathing. Lethality attributed to the given pathogenic load was tested in two animals which met their end points around 78 h post-exposure.

The IACUC approved PO01-08 protocol listed nine clinical parameters (S1 Table); any animal that met 3 or more of those clinical parameters or was moribund, cachectic, or unable to obtain food or water would be humanely euthanized. Based on this guideline, none of the two marked for lethality test did not meet sufficient clinical parameters of euthanasia during the last health checkup. However, within just 2 hours between two successive animal health inspections one animal succumbed to the disease; unfortunately our very competent team did not see the signs of imminent mortality. The other one marked for the lethality test was euthanized at this time after consulting with staff Veterinarian. To note: the NHPs are notoriously able to hide their illness. The pathogenesis of pneumonic plague in this animal was very rapid and even though the veterinary staff took maximum care making observations, the one animal succumbed quickly between the two observation time points (just 2 h). This is true in humans as well and that is why the FDA named the African Green NHP as the “one animal rule” to replicate the progression of illness upon infection with virulent *Y*. *pestis*.

### Bacterial inoculation and aerosol delivery

Cultures of *Y*. *pestis* CO92 strain were grown overnight at 28°C in shaker flasks containing Brain Heart Infusion broth (Becton, Dickinson and Company (BD), East Rutherford, NJ), then diluted directly from the broth to the correct dosage based on spectrophotometric optical density readings (OD600). The suspensions were prepared immediately prior to the administration and kept on ice.

Each of the fasted and anesthetized animals was placed in dorsal recumbency in the plethysmography box, inserting its head through the dental dam head port into the head-only exposure apparatus placed inside a Class 3 biosafety cabinet. The bacteria were nebulized using a low-flow 6-jet Bio Aerosol Nebulizing Generator (BANG, CH Technologies, Inc., Westwood, NJ) with an exposure time of between 5 to 15 m. The aerosol particle stream was delivered at a flow volume of 12 lpm (liter per minute) to each anesthetized monkey which was allowed to breathe freely. Real-time plethysmography (Skornick Plethysmography, CH Technologies, Inc., NJ) was used to measure tidal, minute, and accumulated inhaled volumes during exposure to ensure the correct aerosol dose.

Aerosolized bacteria were sampled from the head exposure chamber into an all-glass impinger containing 20 ml of normal sterile saline (Ace Glass, Inc., Vineland, NJ) and concentrations were confirmed by quantitative bacterial culture. Purity of the aerosolized sample was assessed by colony morphology and growth on Congo Red-containing media. The target particle size, mean mass aerosol diameter (MMAD) of between 0.5 and 3 μm, was determined using a TSI Aerosol Particle Sizer (Model 3321; TSI, Inc., Shoreview, MN) and by side-scatter laser photometry (Microdust Pro; Cassella CEL, Inc., Buffalo, NY). Pathogen dose was calculated using the formula Dose = (C × V), where C was the concentration of viable pathogen in the exposure atmosphere, and V was the total volume inhaled based on plethysmography and exposure times.

### Blood collection, bacteriology and biosample isolation

**Fig 1:**
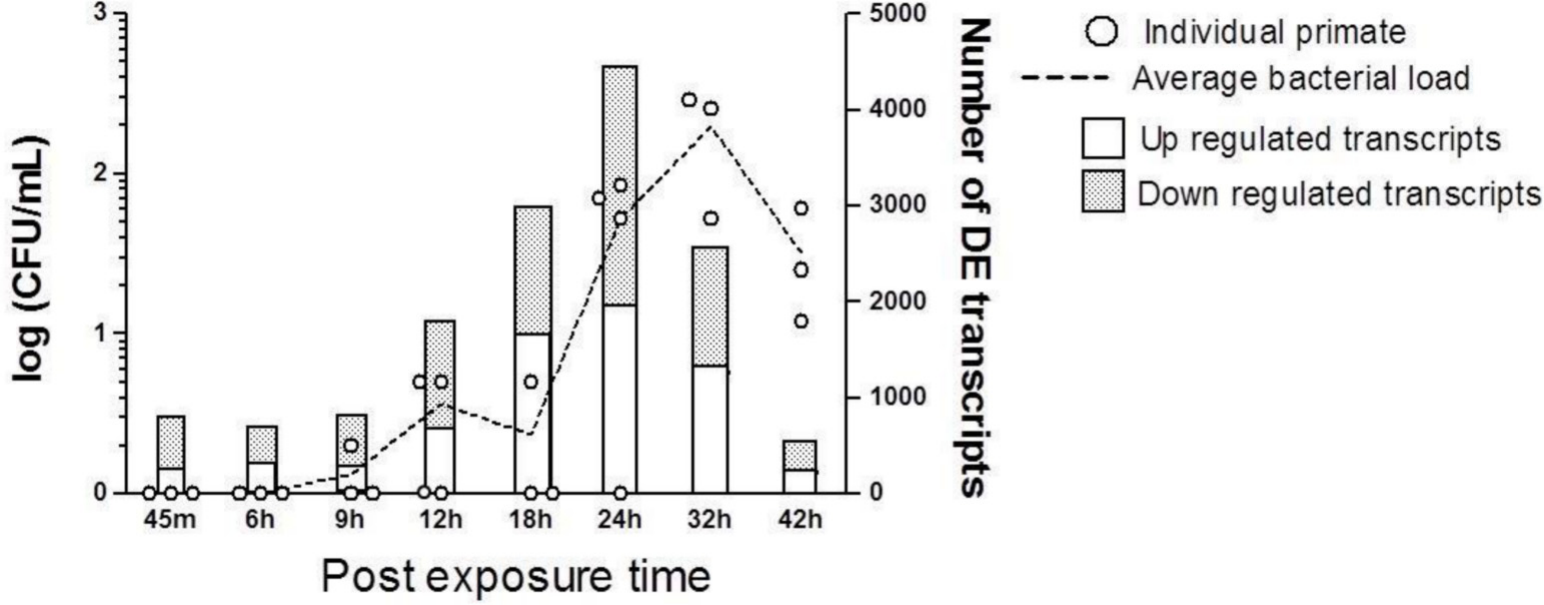
Longitudinal shift of bacteria load in nonhuman primates’ blood and corresponding transcriptional profile. The CFU/mL on the Y-axis (left side) is in log scale. The circles indicate the bacterial load of each animal and the dotted line connects the mean bacterial load across the course of infection. The transcriptomic profile (GoI-*Time*) across the course of infection is also shown on the right side Y-axis. The bars showing the differentially expressed transcripts are divided into up- and down-regulated populations.

From each animal, venous blood was collected twice from the femoral vein (S1 Fig). During each draw, samples were collected into Vacutainer® CPT tubes (BD, Baltimore, MD) and PAXgene tubes (Qiagen, Inc., Germantown, MD). An aliquot of whole blood was plated on a Congo red agar plate and incubated at 28°C for 72 h before counting the colonies. The *in vitro* blood culture results are shown in Fig 1 and S2 Table. At the time of euthanasia, the body temperature of a randomly selected group was recorded. Since, the euthanasia was scheduled till 42 h post-exposure, no body temperature was recorded beyond that time point.

For pathology, the tissues were aseptically collected and portions approximately 1 gm in weight were removed. These were placed into properly labeled, disposable, sterile, sample bags that were then heat-sealed, weighed, and placed on ice. Eventually, these samples were dissociated using a hand-held tissue homogenizer (Tissue Tearor, Inc.) for 120 seconds, serially diluted in sterile saline, and cultured on a Congo red agar plate, and incubated at 28°C for 72 h for quantitative measurement (S2 Table).

Blood collected in CPT tube(s) was centrifuged at 1800 x *g* (approximately 2800 rpm on a Sorvall RT6000 centrifuge) for 20 m at room temperature. Without disturbing the whitish cell layer, the clear plasma from the uppermost layer was transferred to a 15 ml tube and then stored at -80°C. The plasma was used for evaluating the blood chemistry.

For oligonucleotide arrays, whole blood was collected in PAXgene tubes (QIAGEN, MD) and total RNAs were isolated according to manufacturer's instructions, followed by purification using the Min Elute Cleanup Kit (QIAGEN, MD) with the vendor’s protocol. RNA quality and quantity were determined using a Nanodrop ND-1000 spectrometer (Thermo Fisher, Wilmington, DE). RNA from each sample was assessed for purity by *A*_260/280_ ratios. To determine the RNA integrity, we analyzed 1 μl of eluate from each sample with the Agilent 2100 Bioanalyzer according to the manufacturer’s instructions.

### Microarray hybridization and post-processing

Custom Rhesus Monkey Oligo Microarray slides (Catalog number: G2519F-015421) containing ~40,000 probes were obtained from Agilent Technologies (Agilent Technologies, CA). The Agilent Quick Amp Labeling Kit (Agilent Technologies, CA) was used to label BioChain Monkey universal reference RNA (BioChain Institute, Inc., Newark, CA) with Cy3 dye and transcribed mRNA (from either control or *Y*. *pestis* exposed samples) with Cy5 dye. cDNA was hybridized and scanned, and microarray images were visualized and Lowess normalized using Feature Extraction (Agilent Technologies, CA); the resulting data was analyzed using GeneSpring software (Agilent Technologies, CA).

The results are available online (http://www.ncbi.nlm.nih.gov/geo/), GEO ID: GSE63560.

### Biological annotation and functional analysis

Significantly altered transcripts were identified by two methods. Transcripts altered at individual time points (moderated t-test *p* < 0.05) were pooled together and the collection of transcripts was defined as Genes of Interest-*Time Course Differentiation* (GoI-*Time*) (S3 Table and Fig 1 and S2A Fig). This set essentially enlisted those transcripts which were significantly regulated in at least one of the eight time points investigated herein.

Furthermore, we segregated GoI-*Time* into two segments, namely *Early* and *Late* phases of infection based on the chronological order of blood draw. The *Early* phase of infection enlisted all the transcripts significantly altered in any of the 5 time points from 45 m to 18 h. Likewise, the *Late* phase of infection enlisted all the transcripts significantly altered beyond 18 h post-exposure.

Based on the expression dynamics, we also identified two additional subsets from GoI-*Time*, namely GoI-*TimeUp* and GoI-*TimeDown* showing transient pattern of gene expressions correlated with the dynamics of pathogenesis. For this purpose, TimeClust was used for initial filtering (simplification) with Self Organization Map (SOM) and Bayesian Clustering (S4A Fig). SOM is an unsupervised neural network algorithm with prefixed nodes (map-units) displayed on a two-dimensional map [25]. Since the number of genes was very large, we used the SOM approach as a preprocessing step to reduce the dimensionality of the clustering problem; and consequently to reduce the computational burden of the subsequent Bayesian clustering. Bayesian clustering uses a stochastic population model to represent the gene clusters, where each cluster is assumed to be different from the other due to the random effects (individual variability). The default setting of random walk algorithm scripted in TimeClust program [26] was used without changing the value of the highest Bayesian Information Criterion score and without using the heuristic strategy in order to minimize the risk of a suboptimal Bayesian output [27].

Here, GoI-*TimeUp* lists the transcripts showing a gradual pattern of up-regulation during the time course. In this gene cluster, individual gene expressions changed from down-regulation during *Early* time points to up-regulation in the *Late* phase. Contrastingly, GoI-*TimeDown* lists the transcripts showing a gradual trend of down-regulation during the same temporal trajectory. In this gene cluster, individual gene expressions changed from up-regulation during *Early* time points to downregulation in the *Late* phase. S4A Fig shows GoI-*TimeUp* and GoI-*TimeDown* hierarchical clustering computed by Euclidian distance algorithm.

**Fig 2:**
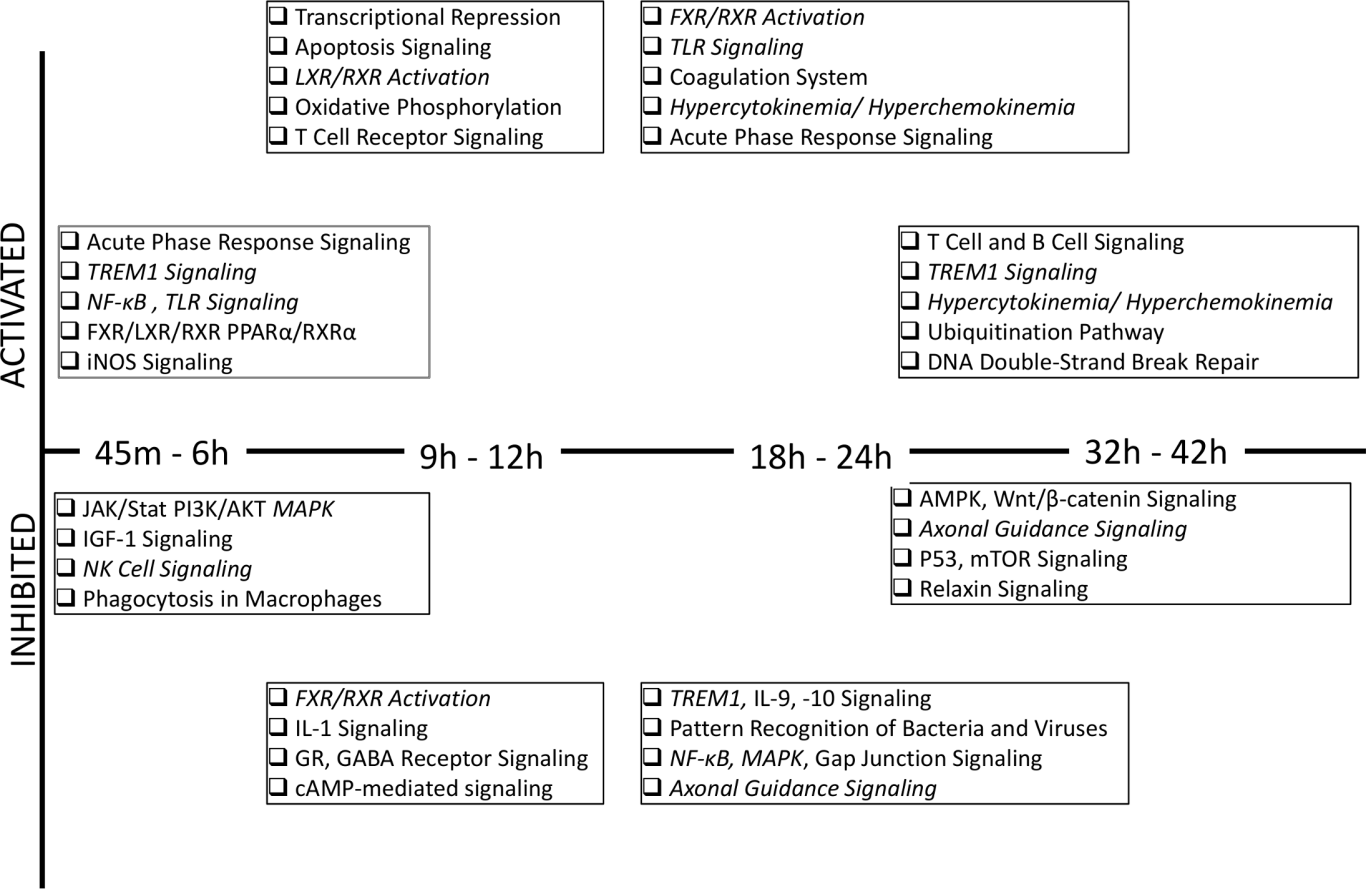
Potentially activated and inhibited GoI-*Time* enriched pathways across the time course. The canonical pathways enriched by up- or down-regulated genes differentially regulated at 45 m-6 h, 9 h-12 h, 18 h-24 h and 32 h-42 h post-exposure are listed. The pathways constituted by the up-regulated genes are listed in the “Activated” section; the pathways constituted by the down-regulated genes are listed in the “Inhibited” section. The functions documented more than once are in italics.

Ingenuity Pathways Analysis (IPA, QIAGEN, Inc., CA) identified those canonical pathways, which were enriched by either up- or down-regulated genomic pool (Fig 2 and S5 Table).

**Fig 3:**
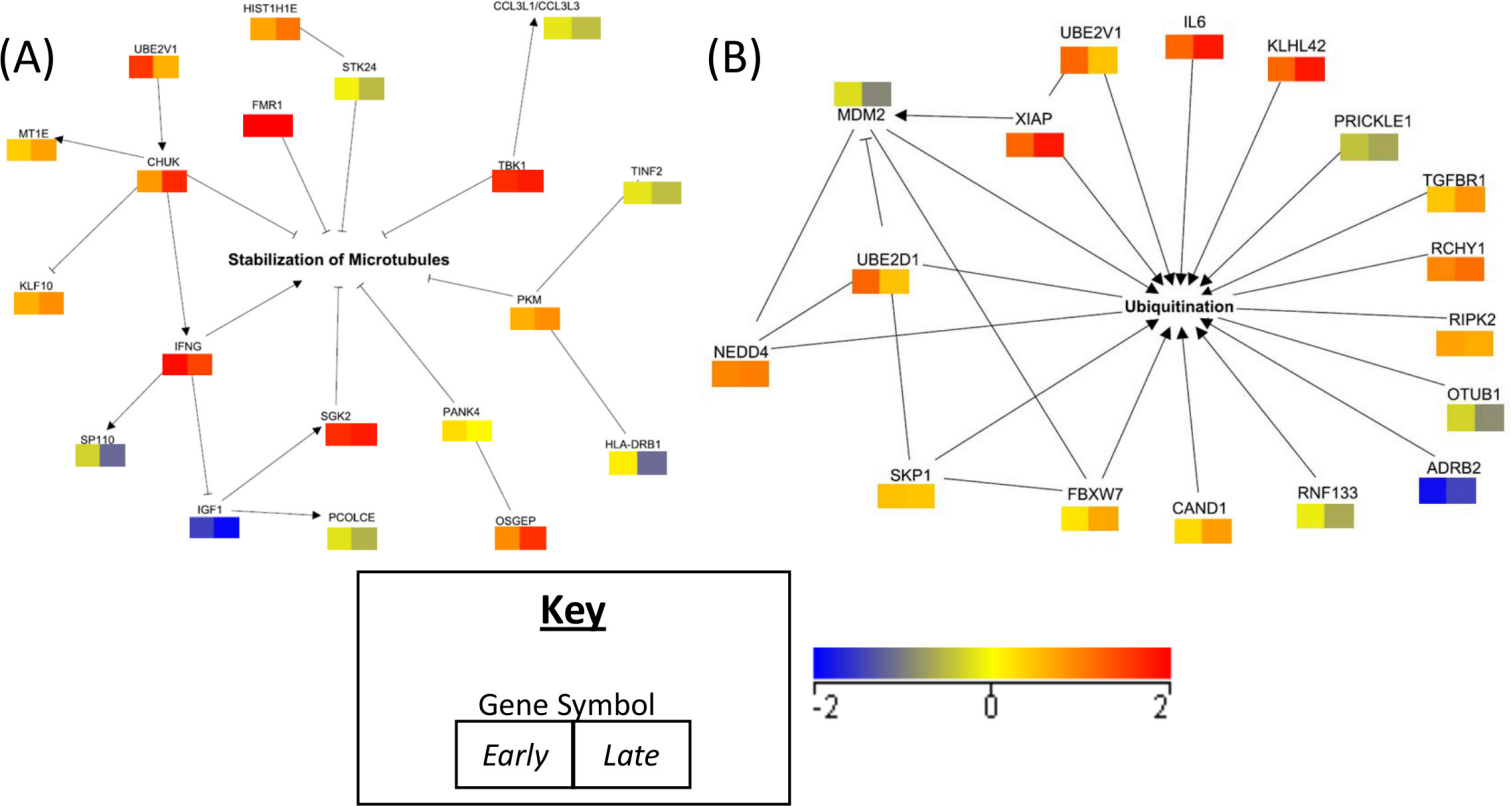
Networks of interest. The average fold changes of Early (45m-18h) and Late (24h-42h) time groups are presented in the right and left boxes, respectively. The color scale presented in the bottom right corner shows the gradient of fold change with red upregulated, blue downregulated and yellow no change in gene expressions. (A) Network of Stabilization of Microtubules. (B) Network profile of Ubiquitination.

In a parallel analysis, we clustered all the blood samples drawn across the time points to form two groups, namely the *Y*. *pestis* exposed group and the unexposed (control) group. The rationale behind this complementary analysis was to identify the molecular signatures associated with *Y*. *pestis* infection irrespective of the time-dependent response. The transcripts significantly altered (moderated t-test *p* < 0.05) between the exposed and control groups were defined as Genes of Interest-Infectious vs. Control (GoI-*Inf*) (S4 Table). S2B Fig shows the hierarchical clustering computed by Euclidian distance algorithm. The Molecular Activity Predictor (MAP) tool of Ingenuity Pathway Analysis (IPA, QIAGEN, Inc., CA) was used to predict the operative modes of the networks, i.e., the activated vs. inhibited status of the networks significantly enriched by GoI-*Inf* (Fig 3 and S3 Fig).

### Quantitative Real-time PCR

**Fig 4:**
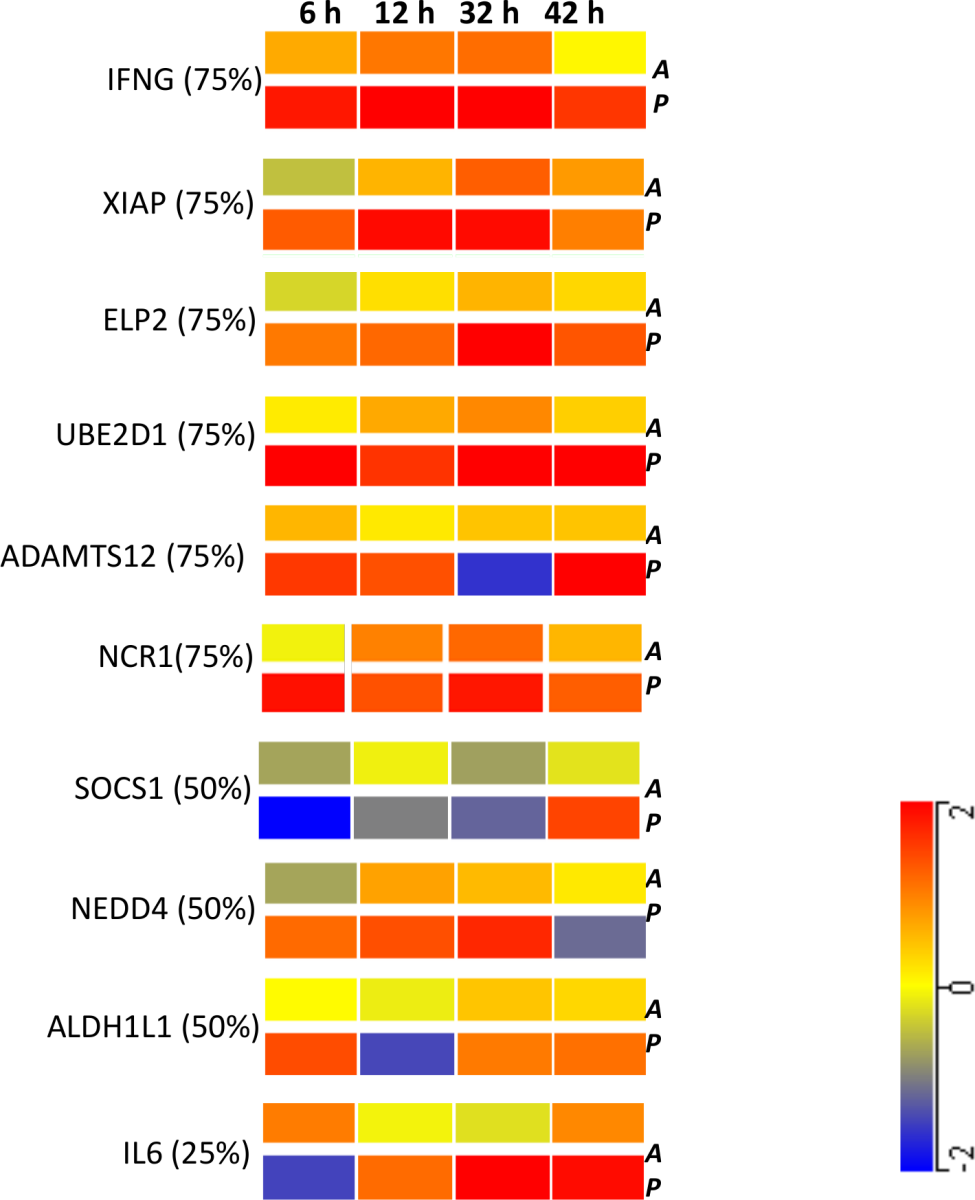
qPCR results of the selected genes arranged in the descending order of validation (% in parenthesis beside the gene name shows the number of genes -fold changed in same direction, >±1.5) between Array (*A*, top row) vs. qPCR (*P*, bottom row) across the time course, two from Early (6 h and 12 h) and two from Late time groups (32 h and 42 h). The color scale presented in the bottom right corner shows the gradient of fold change with red upregulated, blue downregulated and yellow no change in gene expressions.

A panel of genes mined from our microarray result was validated using a real-time quantitative PCR (qPCR) assay as described previously [28]. The selection of these genes was justified by a brief literature survey (Table 1); those genes, which showed involvement with the functions identified in the present study (Results and Discussion of present project) and past findings (listed in the tables), were selected for validation. The primers of the selected genes were available in catalogued assays and were purchased as a custom array plate from SAB Biosciences (Fredrick, MD). qPCR was carried out using the RT^2^ Profiler PCR Array System (SA Biosciences, MD) on the BioRad CFX system (BioRad Laboratories, Inc., Hercules, CA) following manufacturers’ instructions. Two housekeeping genes (ACTB and GAPDH) were used for normalization. Data was analyzed using the Excel-based RT^2^ profiler PCR Array Data Analysis using the 2^∆∆ct^ method (Fig 4). qPCR outputs were compared to the array results. We considered these values mutually validated if the outcomes from the two assay platforms showed regulation in same direction, i.e., either both up- or down-regulated with -fold change greater than ±1.5.

**Table 1:** A brief literature survey underlining the relevance of the genomic pool selected for qPCR.

Gene Symbol/ Name	Description
IFNG/ Interferon gamma	In the mouse pneumonic plague model, IFNG activation was observed as the consequence of pathogenesis [9].
	At the transcriptomic level, IFNG was elevated in mouse models with exposure to *Y*. *pestis* strain of CO92 [11], but suppressed with exposure to an avirulent strain [49].
XIAP/ X-linked inhibitor of apoptosis protein	*In vitro* retroviral infection mediates XIAP to trigger the NF-κB signaling pathway as a pro-survival loop and to inhibit cell proliferation [50].
ELP2/ Elongator Acetyltransferase Complex Subunit 2	This Elongator subunit is intricately associated with chromatin remodeling [34] and mediates the pro-survival and pro-apoptotic pathways [51].
UBE2D1/ Ubiquitin-conjugating enzyme E2 D1	Yersinia virulence factor interacts with UBE2D1 to suppress the NF-κB signaling pathway, and thereby inhibits the downstream proinflammatory activities [52].
ADAMTS12/ ADAM Metallopeptidase With Thrombospondin Type 1 Motif, 12	ADAMs play key roles in initiating or terminating immunological processes; furthermore, they assist in cytokine biosynthesis and direct systemic inflammation or barrier immunity [53].
NCR1/ Natural cytotoxicity triggering receptor 1	Initiating natural killer (NK) cell-mediated cytolysis, NCR1 is considered a typical marker for NK cells [54].
SOCS1/ Suppressor of cytokine signaling 1	SOCS1 regulates the cytokine signaling via negative feedback loop; hence, it plays a critical role in combating endotoxic challenges [55].
NEDD4/ E3 Ubiquitin-Protein Ligase	NEDD4 enhances ubiquitination and proteolytic degradation of many members of the toll-like receptor channel, and thereby controls the early pathogenic recognition process [46].
ALDH1L1/ Aldehyde Dehydrogenase 1 Family, Member L1	Overexpression of ALDH family members helps to ameliorate oxidative stress [56].
	ALDH family members control cellular motility [57].
IL6/ Interleukin 6	*In vivo* exposure of LPS surges the IL-6 load in equine whole blood [58].
	A study of the patients in surgical units found IL-6 as an early tool to diagnose severe sepsis [59].

### Blood Chemistry

**Fig 5:**
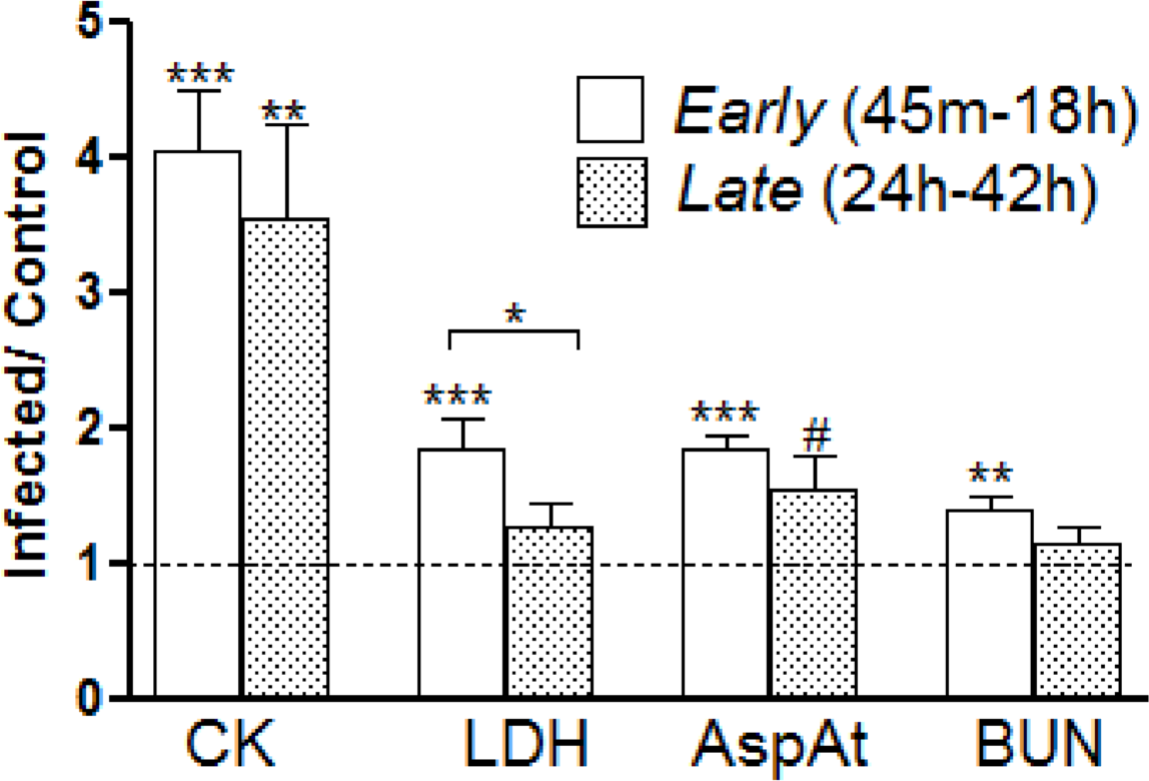
Longitudinal profile of plasma chemistry. The expressions of selected chemicals (Exposed/Controls) are shown. The values measured from 45 m to 18 h were pooled together as the *Early* group (white bar) and those from 24 h to 42 h were clustered as the *Late* group (shaded bar). The horizontal broken line depicts the control value calculated from the blood samples drawn 24 h before aerosol exposure. Welch’s t-test computed: # 0.1 < *p* < 0.05, * *p* < 0.05, ** *p* < 0.001, *** *p* < 0.0001.

Plasma samples obtained from the infected and uninfected nonhuman primates (NHP) were assessed for chemical parameters. These include: glucose, blood urea nitrogen (BUN), creatinine, sodium, potassium, chloride, carbon dioxide, calcium, phosphorus, cholesterol, triglycerides, total protein, albumin, aspartate aminotransferase (Asp At), alanine transaminase (ALT), lactate dehydrogenase (LDH), creatine kinase (CK), alkaline phosphatase (ALKP), gamma-glutamyl transpeptidase (GGT), and total bilirubin. In order to ensure safe transfer of the samples out of the BSL-III facility, the plasma was first filtered through a 0.2 micron filter, followed by screening of the samples for bacterial growth. Once all samples were detected as pathogen-free, 300 μl samples were analyzed (Fig 5). The blood drawn 24 h before the aerosol challenge was the baseline (dotted horizontal line, Fig 5), which was pairwise (Welch’s t-test *p* < 0.05) contrasted against two post-exposure clusters, namely *Early* (45 m-18 h) and *Late* (24 h-42 h).

## Results

Twenty-six African green monkeys were subjected to aerosol challenge with *Y*. *pestis* strain CO92. Particular attention was paid to ensure equivalent delivery of pathogenic load. Blood was drawn 24 h before the exposure (baseline control) and at eight chronologic time points (45 m, 6 h, 9 h, 12 h, 18 h, 24 h, 32 h and 42 h) post-exposure. Three control animals were sham-exposed to saline under identical conditions similar to that of *Y*. *pestis* exposure. Healthy rearing of those animals in parallel to the exposed animals attested to the sanitation of their housing environment. In addition, two infected controls were reared for lethality test meeting end points 78 h post-exposure. The average MMAD ± geometric standard deviation of the generated aerosol particles was 1.03 μm ± 1.46, and based on our calculation, the inhaled *Y*. *pestis* exposure for each animal was in a range of exposures between 0.33 x 10^6^ and 3.55 x 10^6^ CFU.

The quantitative measurements of *Y*. *pestis* present in the exposed animals’ blood and urine samples and nine tissues, including axillary lymph node, submandibular lymph node, inguinal lymph node, mediastinal lymph node, mesenteric lymph node, spleen, liver, lung and kidney, were evaluated (Fig 1 and S2 Table) based on the geometric mean of CFU/ml of the dilution factor and colony counts. Bacterial colonies were detectable at 9 h post-exposure among a subset of the infected cohort. Some of the results reported herein failed to pass the threshold value (> 200 CFU/ml); nevertheless, they are presented, since we noticed viable colonies of the inoculated bacteria. Notably, we found no correlation of the bacterial enumeration in blood with the inhaled dose for individual NHPs. Bacteria were detected in blood 9 h after exposure in 1 of 3 animals, 2 of 4 at 12 h, 1 of 3 at 18 h, 3 of 4 at 24 h, 3 of 3 at 32 h, and 3 of 3 at 42 h. Even so, liver and lungs showed very early and consistent load of infection from 6 h post-exposure.

Regarding other pathological data, we noticed minimum changes in the body temperature between 6 h and 42 h post-exposure with a range of 34.2°C to 37.3°C and the average temperature was 36.01±0.8°C. Much of the temperature differences were attributed to the individual variances; maximum temperature change in individual NHP recorded in this study was 1.3°C with average change in individual NHP was only 0.18°C. Those are in agreement with an earlier report [12].

### Transcriptomic analysis

In comparison with the baseline defined by the 24 h pre-exposed blood samples, the differentially regulated transcripts at the post-exposure time points were identified using moderated t-test (*p* < 0.05). Fig 1 and S2A Fig show that the number of significantly differentially expressed transcripts gradually increased from 45 m post-exposure, reaching a maximum during 18 h and 24 h, with a subsequent decrease. Together, there were 10,059 unique transcripts enlisted by GoI-*Time*, but no transcripts were found conserved across all the time points investigated herein.

Furthermore, GoI-*Time* (S2A Fig and S3 Table) was segregated in chronologic order. As explained earlier, *Early* phase of infection covered from 45 m to 18 h post-exposure time points, while the *Late* phase of infection covered the remainder, starting from 24 h post-exposure. Making the demarcation between 18 h and 24 h was justified by the bell-shaped transcriptional expression profile (Fig 1 and S2A Fig), which peaked between 18 h and 24 h. There were 6,495 and 6,020 transcripts altered during the *Early* and *Late* phases of infection, respectively, while 2,456 transcripts were conserved between these two phases of infection. Using the SOM algorithm, we further sorted GoI-*Time* to mine the transcripts showing consistent temporal changes either towards up-regulation (138 transcripts) or down-regulation (225 transcripts) (S4 Fig) designated as GoI-*TimeUp* and GoI-*TimeDown*, respectively.

In a parallel analysis, we identified 1,274 transcripts defined as GoI-*Inf* (S2B Fig and S4 Table) that were differentially regulated between the *exposed* and *baseline* values; the latter was derived from the blood drawn 24 h pre-exposure. GoI-*Inf* was essentially the collection of all the transcripts identified in the blood samples drawn across the entire time course post *Y*. *pestis* exposure. Interestingly, a considerable number of GoI-*Inf* transcripts (82%*)* were found conserved with GoI-*Time* (S2C Fig).

### Functional analysis of GoI-*Time*

A brief overview of the canonical functional analysis of GoI-*Time* is depicted in Fig 2 and the details are listed in S5 Table. In addition, parallel functional analyses of GoI-*TimeUp* and GoI-*TimeDown* were performed to elucidate time-dependent modifications of the functional activities. S4B Fig depicts the hierarchical clustering of 25 transcripts from GoI-*TimeUp* associated with the most enriched functional modules. These genes were further clustered into three sub-groups (A_*up*_, B_*up*_ and C_*up*_) based on their biological interactions mapped from literature mining. S4B Fig also displays the hierarchical clustering of 86 transcripts from GoI-*TimeDown* associated with the most enriched modular interaction networks. The corresponding networks are shown in S4C, S4D Fig.

### Functional analysis of GoI-*Inf*

Functional annotation of GoI-*Inf* (S3 Fig) demonstrated a significant enrichment in apoptosis (S5A Fig), stabilization of microtubules (Fig 3A), recruitment of inflammatory leukocytes (S5B Fig), formation of cellular inclusion bodies, synthesis of extracellular matrix, and myocardial and liver failure. Protein ubiquitination signaling (Fig 3B), G2/M DNA damage checkpoint, growth hormone signaling, and p53 signaling pathways were the top enriched canonical pathways.

The activation or inhibition dynamics of the networks across the infection period were predicted by MAP (IPA, CA) with the relationships scored *in silico* based on the regulations of the molecules connected to the relevant annotation (S3 Fig). MAP predicted a consistent activation of apoptosis encompassing 47 transcripts. Fourteen and eight apoptotic transcripts were associated with DNA repair and chromatin condensation, respectively, although with a limited predictive power due to small sample size. MAP further predicated temporally consistent activation of the recruitment of inflammatory leukocytes and ubiquitination, and potential inhibition of the microtubule stabilization across the time course.

### Validation using qPCR results (Fig 4)

The high throughput microarray data was validated by real-time qPCR. A brief literature survey outlining the relevance of the ten genes selected for this study is described in Table 1. For the qPCR study, two time points were selected from *Early* (6 h and 12 h) and *Late* (32 h and 42 h) phases of infection, respectively. Fig 4 depicts the comparative analysis of the oligonucleotide microarray result and the qPCR result. The number of transcripts showing similar regulations in both platforms was calculated. Six transcripts showed similar expression in three out of four time points, and four transcripts showed similar -fold changes in two out of four time points.

### Blood Chemistry (Fig 5)

The blood-based evaluation was limited to the blood chemistry of the serum. A detailed protocol was described that highlights our effort to maintain the serum pathogen-free. For safety reasons, we could not inspect the basic cellularity of blood potentially carrying BSL-3 pathogens. It was a drawback of this study.

To compute the blood chemistry, we clustered the exposed animals into two groups in an attempt to enhance the statistical power of the analysis. As noted previously, the *Early* phase of infection consisted of the samples collected over the time course from 45 m to 18 h, whereas the *Late* phase of infection consisted of the samples collected from 24 h to 42 h. Comparisons of the *Early* or *Late* phase of infection versus the baseline controls (pre-exposure blood) showed CK and AspAt consistently elevated. BUN and LDH were significantly elevated in the *Early* phase of infection, but these returned to the baseline during the *Late* phase of infection.

## Discussion

We studied the longitudinal dynamics of perturbation of the blood transcriptome of the African green monkey (*Chlorocebus aethiops*) as a consequence of aerosol exposure to *Y*. *pestis*. The baseline of the study was defined by the blood samples drawn 24 h before exposing the monkeys to *Y*. *pestis*; the post-exposure blood samples were drawn at eight sequential time points (S1 Fig). Two of the treated animals were allowed to live past 42 h and met end points at nearly 78 h post-exposure.

The lethality test showed that the pathogen load used in the present study, if left untreated, can cause death within 78 h post-exposure. Therefore, to characterize the early dynamics of pathogenesis, we investigated the transcriptomic profile till the half-way through the survival curve, which was approximately 42 h post-exposure.

The animals showed no elevation of body temperature till 42 h post infection. No viable bacterial presence was recorded until 9 h post-exposure; in fact, only a fraction of infected NHPs manifested bacterial colonization in the blood prior to 24 h post-exposure, which was in agreement with the previous report [12]. Further investigation found bacterial loads in kidney, lungs and liver at much earlier time points (S2 Table). It could be argued that the pathogens were possibly nested inside and transported via the macrophages and other blood cells during this *Early* phase of infection, thereby evading *in vitro* detection in blood [11]. Other possibilities such as potential delayed emergence of bacteria from the pulmonary tract and/or the lymph nodes could not be overruled, and this underlines the necessity of further investigation.

The molecular perturbations were noted at 45 m post-exposure (Fig 1), much earlier than the emergence of the bacterial colonies in blood; although we cannot overrule the potential contributions of certain external factors, such as anesthesia, in changing the early genomic profile. In addition, this study presented the transcriptomic regulation averaged over all the blood cell types. Cell-specific data could produce a wealth of information, but this was beyond the scope of the present approach.

Oligonucleotide microarray data was analyzed in two complementary fashions. First, we probed the transcriptomic profile at individual post-exposure time points (GoI-*Time*). Second, we contrasted the entire infected cohort, irrespective of their infection duration, against their baseline derived from the blood drawn 24 h pre-exposure (GOI-*Inf*). It is important to note that the present result is limited by probing the Rhesus Macaques (Macaca mulatta) cDNA array, but rhesus macaque was phylogenetically diverged from African Green Monkey 10–15 million years ago. This phylogenetic divergence could bias the results regarding the most rapidly-evolving immune gene families, such as variable cell-surface ligands or variable receptors; some of those have been identified in the present study. To mitigate the potential bias due to the evolutionary divergence, a next generation sequencing study is recommended.

Characterizing GOI-*Time*, we observed that the number of transcripts gradually increased until 24 h, followed by a steady reduction. The initial rise of transcriptomic numbers was potentially due to the accumulative influences of the pathogenic infection. However, at later phases of infection, simultaneous activation of many intertwined networks potentially triggered conflicting feedbacks to the transcriptional activities. As a result, the number of differentially expressed genes was reduced among the moribund animals. Similar temporal distribution of the genomic profile depicting a bell-shaped curve was observed previously in a study of *Mycobacterium tuberculosis in vivo* [29].

GoI-*Time* elucidated valuable information about the molecular shift as the function of the time lapse after the pathogenic challenge. This strategy was, however, particularly vulnerable to the limited statistical power. The sample size of each time point was limited to three or four. Therefore, we complemented the analysis with GoI-*Inf* as the genomic profile of all post-exposed 26 NHPs were clustered, irrespective of their infection durations, and contrasted against the baseline derived from the blood drawn 24 h pre-exposure. With this larger sample size, we expected a better statistical confidence in elucidating the pathogenic implications over the entire infection period while at a cost of the finer detail of time-specific information. Interestingly, around 83% of the transcriptomic members of GoI-*Inf* were also conserved in GoI-*Time*, and they were almost equally distributed between the two *Early* (45 m – 18 h) and *Late* (24 h—42 h) phases of infection.

A systematic modulation of the host immunity initiated at the onset of infection was evident from transcriptomic results. Supporting a previous study [8], *Y*. *pestis* first suppressed the genes enriching networks associated with the natural killer (NK) cell signaling and phagocytosis in macrophages to overwhelm the innate immunity (Fig 2). A pool of pro-inflammatory transcripts, such as SOCSS1, UBE2D1, and ADAMTS12, was differentially regulated as shown by qPCR results (Fig 4). Many cytokines, such as IL-1A, -11, -18, and -27, and chemokines, such as CCL-1, -14, -17, and -26, and CXCL16 showed gradual transcriptomic suppression. Early onset of apoptosis manifested by the early perturbation of JAK/Stat, PI3K/AKT and MAPK pathways (Fig 2) potentially played a significant role in facilitating *Y*. *pestis* evasion of early pathogenic recognition [30,31]. In fact, the active toll-like receptor (TLRs) signaling networks (Fig 2) possibly failed to contain *Y*. *pestis*. Further studies are necessary to confirm this hypothesis.

Delayed suppression of pathways linking MAPK and NF*κ*B signaling potentially triggered rapid apoptosis or necrosis at the later time points (S3 Table) [32]. In addition, mTOR, AMPK and p53 signaling networks were also suppressed beyond 24 h post-exposure (S3 Table). Those potentially facilitated the pathogen-promoted annihilation of the host defense [33]. As a consequence, two biofunctions closely linked with apoptosis, namely the repair of DNA and chromatin condensation, were suppressed. The qPCR result reported the consistent up-regulation of ELP2, a mediator of the chromatin remodeling process [34].

In a parallel chain of events in the *Early* phase of infection, activation of many inflammatory pathways, such as FXR/RXR, TREM, iNOS and LXR/RXR triggered the cytokine production in macrophages and oxidative phosphorylation (Fig 2). The increasing expression of TLR molecules, though failing in pathogenic recognition, potentially activated NF*κ*B and thereby initiated the pro-survival loop [35]. Subsequent elevation of the genes encoding the inflammatory chemokines such as CCL16, CCL25 and CXCL6, and cytokines such as IL-2, IL-8 and IFNG (Fig 4) in concert with delayed activation of the coagulation system (Fig 2) are the typical signatures of sepsis [36] [37].

With the first indication of an uncontrolled surge of cytokines and chemokines (activated networks of hypercytokinemia and hyperchemokinemia, as shown in Fig 2), 24 h seemed to be the time point when *Y*. *pestis* began to overwhelm the host defense mechanisms. Beyond the 24 h post-exposure, *Y*. *pestis* began to perturb many cancer-related networks affecting organs such as pancreas, breast, lungs, liver and bone marrow (S5 Table). An uncontrolled surge of cytokines and chemokines was suggested beyond 24 h post-infection, which, coupled with multi-organ failures, could bring about fatality in rapid succession [38].

Supportive of past evidence [39], the present report also indicates that *Y*. *pestis* infection potentially delayed the activation of adaptive immunity. The networks associated with T-cell and B cell-activation were triggered beyond the 24 h post-exposure (Fig 2). These late responses were likely to be ineffective in containing the already well-proliferated pathogenesis. The present study, in concurrence with many previous observations [40–42], suggests value in including priming of cellular immunity in the next generation vaccination program.

The longitudinal dynamics of the transcriptomic shift (Fig 1) suggestively contradicts the biphasic *Y*. *pestis* progression pattern reported in the mouse model [9,22], which portrayed an early phase of subdued recruitment of inflammatory markers turning into the uncontrolled discharge of cytokines and chemokines. However, a recent study showed an early response of a very different quality of cytokines in a potentially plague-resistant strain of mouse [19]. Extending the knowledge, the present NHP model demonstrated an *Early* pro-inflammatory phase evidently mediated by active ubiquitin and lymphocytes, which eventually turned into hypercytokinemia and hyperchemokinemia.

In the context of early immune response, we draw attention to our observation of hematological chemistry (Fig 5). The early spike of BUN and LDH load potentially reflects the cytokine-mediated muscular catabolism [43] and cytotoxicity [44] ensuing immediately after the *Y*. *pestis* exposure. The early elevation of CK and AspAt possibly gives an estimation of the scale of rapidity with which *Y*. *pestis* was disseminated to liver and muscle tissues. These observations along with the bacterial load enumeration matrix (S2 Table) potentially support the hypothesis that the pathogens were transported via macrophages and other blood cells during the *Early* phase of infection, and thereby escaped early detection (Fig 1) [11]. Comprehensive transcriptomic investigation focused on the tissues of interest is, therefore, crucial in order to understand the route of pathogenesis and the pertinent mechanisms.

The role of microtubules in *Y*. *pestis* uptake via epithelial cells was suggested previously [45]. Epithelial cells served as an essential port for the inhalation exposure in the present study. Therefore, the genes encoding the early destabilization of the microtubules (Fig 3A) could have therapeutic implications. Furthermore, longitudinal annotations of the transcriptomic profile predicted a consistent elevation of ubiquitination (Fig 3B). qPCR results showed early overexpression of NEDD4, a genomic activator of ubiquitination [46]. The contribution of ubiquitination to cellular immunity [47], particularly in mediating NFκB activation [48], could be explored further in the quest for an effective therapy against *Y*. *pestis*.

## Conclusions

The transcriptomic analyses suggest a very rapid molecular perturbation in blood was caused by *Y*. *pestis* assault in African green monkeys. The host defense was essentially exhausted, and the process of multi-organ failure was initiated only one day after the aerosol exposure, while the body temperatures showed no elevation. Therefore, we speculate that the molecular signatures mined prior to the 24 h post-exposure could have early therapeutic potential.

During the early phase of pathogenesis, systematic modulation of both adaptive and innate immunity programs was mediated in parallel with activation of toll-like receptor-based pathogenic detection. Despite that, pathogenesis progressed unabated. We hypothesize that the early onset of apoptosis facilitated the disease proliferation [30,31]. This hypothesis is subject to verification through an independent study.

We also observed early inhibition of natural-killer cell signaling networks causally compromising the innate immunity, in agreement with previous observations [39]. The delayed activation of adaptive immunity would likely to come out ineffective in combating highly-proliferating pathogens. Coordinated priming of innate and adaptive immunity is thereby suggested as a potentially effective method for next generation vaccination programs [40–42]. In the context of immunological health, the involvement of ubiquitin in *Y*. *pestis* pathogenesis is worth exploring in the future.

African green monkeys showed an early surge of pro-inflammatory immune response via activation of lymphocytes, which was a possible sign of deviation from the biphasic *Y*. *pestis* pathogenesis model typically reported in rodents [9,22]. Since the present NHP model is phylogenetically closer to human, our observations could be very important in defining next generation care; however, further validation is necessary.

Along this line, we also must admit that we found a number of similarities between our NHP model and previously published murine models. Many of the genes identified in the present study, such as IL2RG, NdRG, IFNG, and CXCL6, were also regulated in the mouse pneumonic plague models [15,49]. Increased IFNG and IL-6 loads were also reported in *Y*. *pestis*-infected rodent lungs [9]. Furthermore, many networks identified in the present study are in agreement with the murine tissue model [11], namely the networks linked to the antigen presentation, cell death, cell-mediated immunity, inflammatory response, hematopoiesis, acute phase response, glucocorticoid receptor signaling, DNA repair, free radical scavenging and LXR/RXR activation.

In summary, the present study recognizes a potential pattern of *Y*. *pestis* pathogenesis in evasion of the early host defense mechanism. The role of microtubules, ubiquitination, and the contribution of early apoptotic aggravation suggested in this context are subject to further validation. Follow-up comprehensive investigations focused on organs of interest are underway to further elucidate the pathogenesis and disease proliferation.

## Supporting Information

S1 Fig Flow chart of the study design. The study began with initial blood draws conducted at *T*_*0*_−24 h. At *T*_*0*_, nonhuman primates were exposed to *Y*. *pestis* (marked as EXP+). Final blood draws were carried out at eight different time points marked as *T*_*0*_ + 45 m, 6 h, 9 h, 12 h, 18 h, 24 h, 32 h and 42 h. The number of primates (*N*) used for each time point is indicated as well. All of the animals except the 45 m cohort were euthanized to collect organs stipulated for another study. The same cohort of animals was bled twice, at 45 m and 6 h post-stress.
(TIF)

S2 Fig Comparative analysis of GoI-Time and GoI-Inf. The transcriptomic profile of GoI-Time: across the time course. The volcano graph is a plot of p-value [-log10(p-value)] against -fold change [log2(fold change)]. (B) Hierarchical clustering of GoI-Inf across the time course. The color scale presented in the bottom right corner shows the gradient of fold change with red upregulated, blue downregulated and yellow no change in gene expressions. (C) Relationship between GoI-Inf and GoI-Time transcripts. The Venn diagram shows the overlap of transcript populations between Gol-Inf and the two sets of GoI-Time. These two subsets of GoI-Time are the following: (i) the Early group is a pool of the transcripts significantly altered at 45 m, 6 h, 9 h, 12 h and 18 h; (ii) the Late group is a pool of the transcripts significantly altered at 24 h, 32 h and 42 h.
(TIF)

S3 Fig Longitudinal dynamics of functions enriched by GoI-*Inf*: The functional regulations were computed by Molecular Predicative Analysis (MAP), a z-score-based algorithm provided by IPA. The up- and down-wards arrowheads depict the activated and inhibited levels of these functions. The non-active levels are shown by ‘X’. The functions of interests are (i) Apoptosis, (ii) Stabilization of microtubules, (iii) Recruitment of inflammatory leukocytes, (iv) Ubiquitination, (v) DNA repair, and (vi) Chromatin condensation.
(TIF)

S4 Fig Characterization of GOI-*TimeUp* and GoI-*TimeDown*. (A) Hierarchical clusters of two subsets of GoI-*Time* showing a significant gradual trend of shifting towards (i) overexpression (GoI-*TimeUp*) and (ii) suppression (GoI-*TimeDown*). The gene sets were named GoI-*TimeUp* and GoI-*TimeDown*, respectively. The color scale presented in the bottom right corner shows the gradient of fold change with red upregulated, blue downregulated and yellow no change in gene expressions. (B) Figure S4(B). Characterization of GOI-*TimeUp* and GoI-*TimeDown*. Hierarchical clustering of the focus list from GoI-*TimeUp* and GoI-*TimeDown*: The left hierarchical cluster depicts the subsets from GoI-*TimeUp* showing the most enriched modular interactions. They are grouped in three sub-clusters (A_up_-C_up_) and the related functions are listed (in bullets) to its right. During the infection lifecycle, these functions were consistently enriched by gradually overexpressing genes. The right hierarchical cluster depicts the subsets from GoI-*TimeDown* showing the most enriched modular interactions. They are grouped in fives (A_down_ -E_down_) and the related functions are listed (in bullets) to its right. During the infection lifecycle, these functions were consistently enriched by gradually suppressing genes. The color scale presented in the bottom right corner shows the gradient of fold change red upregulated, blue downregulated and yellow no change in gene expressions. (C) Network of the focus subset from GoI-*TimeUp*. As shown in Figure S4B, the genes are clustered into the group identified as A-C_up_.(D) Network of the focus subset from GoI-*TimeDown*. As shown in Figure S4B, the genes are clustered into the groups identified as A-E_down_.
(TIF)

S5 Fig Networks of interest. The candidate genes are colored by their regulation at 24 h. The color scale presented in the bottom right corner shows the gradient of fold change with red upregulated, blue downregulated and yellow no change in gene expressions. (A). Network of the apoptosis profile. (B). Network of the recruitment of inflammatory leukocytes.
(TIF)

S1 Table The lethality endpoints approved under IACUC approved protocol PO01-08: Systems biology studies to identify host indicators /therapeutic targets at early time periods post exposure to Y. pestis (CO92) in African Green Monkeys (Chlorocebus aethiops). Any animal found to present with 3 or more of the clinical signs or to be moribund, cachectic, or unable to obtain food or water will be judged to be at or near the endpoint and humanely euthanized
(XLSX)

S2 Table Bacterial load enumerated from different organs of infected animals. *Not applicable; **No animals were euthanized at 45m; therefore no bacterial loads were detected in tissues. These same animals were euthanized at the 6h time point. ***One animal died at 72h and the other one was euthanized at 78h; so all the values in bacterial loads were enumerated at 78h euthanasia time points.
(XLSX)

S3 Table The GoI-*Time* regulation profile. **This is the list of genes significantly regulated at individual time points.** The values are in log_2_(fold change).
(XLSX)

S4 Table The GoI-*Inf* regulation profile. This is the list of genes identified by contrasting all the post-exposure samples (irrespective of time of infection) vs. the respective controls (bloods drawn before the aerosol challenge). The values are in log_2_(fold change).
(XLSX)

S5 Table The canonical pathways enriched by GoI-*Time*. (XLSX)

S6 Table Genes associated with the functions significantly enriched by GoI-*Inf*. The values are in log_2_(fold change).
(XLSX)
