# Correction: Temporal Progression of Pneumonic Plague in Blood of Nonhuman Primate: A Transcriptomic Analysis

**DOI:** 10.1371/journal.pone.0154006

**Published:** 2016-04-14

**Authors:** Rasha Hammamieh, Seid Muhie, Richard Borschel, Aarti Gautam, Stacy-Ann Miller, Nabarun Chakraborty, Marti Jett

The sixth author, Nabarun Chakraborty, should be noted as contributing equally to this work.
