# Supplementary figures and images for: Temporal Progression of Pneumonic Plague in Blood of Nonhuman Primate: A Transcriptomic Analysis
Source: PLoS One. 2016 Mar 22;11(3):e0151788. doi: 10.1371/journal.pone.0151788 (PMC4803270; doi:10.1371/journal.pone.0151788)

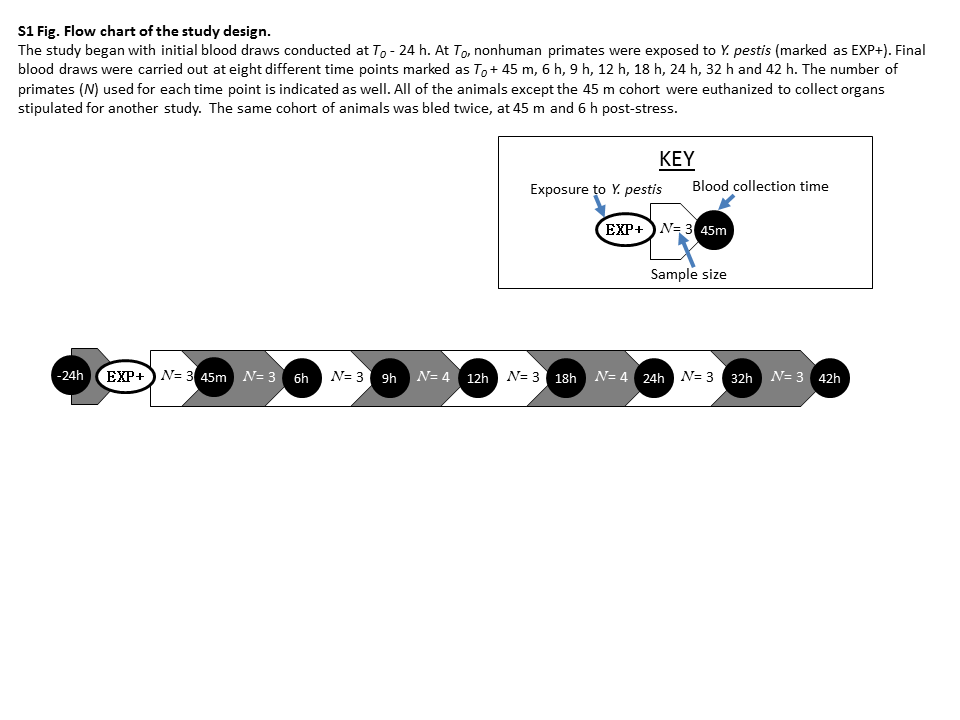

Supplement: S1 Fig — The study began with initial blood draws conducted at T0−24 h. At T0, nonhuman primates were exposed to Y. pestis (marked as EXP+). Final blood draws were carried out at eight different time points marked as T0 + 45 m, 6 h, 9 h, 12 h, 18 h, 24 h, 32 h and 42 h. The number of primates (N) used for each time point is indicated as well. All of the animals except the 45 m cohort were euthanized to collect organs stipulated for another study. The same cohort of animals was bled twice, at 45 m and 6 h post-stress. (TIF) [file pone.0151788.s001.TIF]

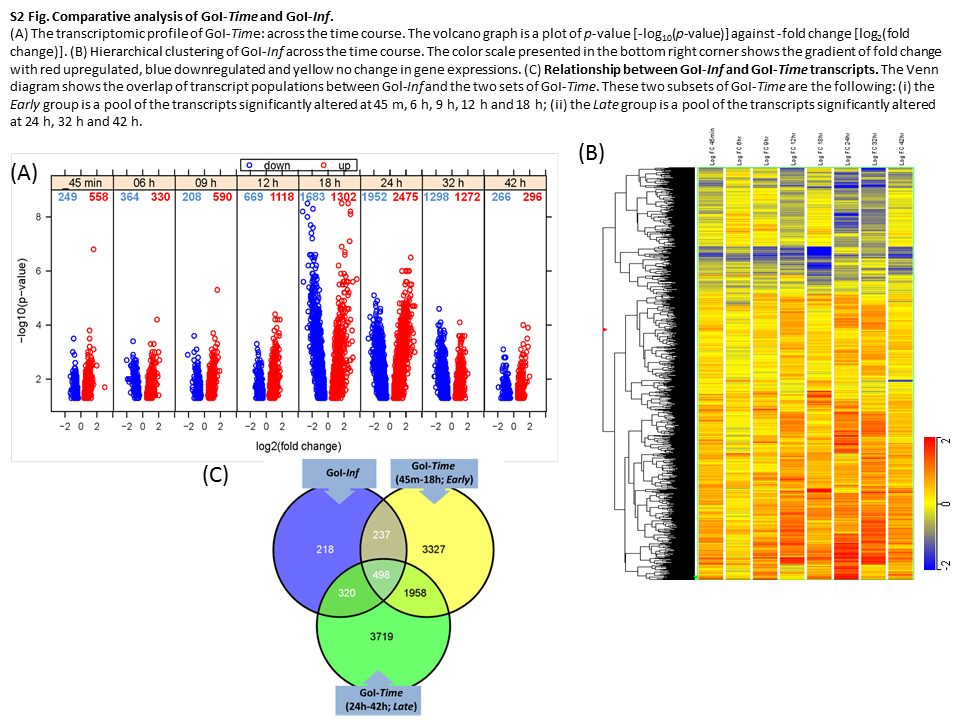

Supplement: S2 Fig — The transcriptomic profile of GoI-Time: across the time course. The volcano graph is a plot of p-value [-log10(p-value)] against -fold change [log2(fold change)]. (B) Hierarchical clustering of GoI-Inf across the time course. The color scale presented in the bottom right corner shows the gradient of fold change with red upregulated, blue downregulated and yellow no change in gene expressions. (C) Relationship between GoI-Inf and GoI-Time transcripts. The Venn diagram shows the overlap of transcript populations between Gol-Inf and the two sets of GoI-Time. These two subsets of GoI-Time are the following: (i) the Early group is a pool of the transcripts significantly altered at 45 m, 6 h, 9 h, 12 h and 18 h; (ii) the Late group is a pool of the transcripts significantly altered at 24 h, 32 h and 42 h. (TIF) [file pone.0151788.s002.TIF]

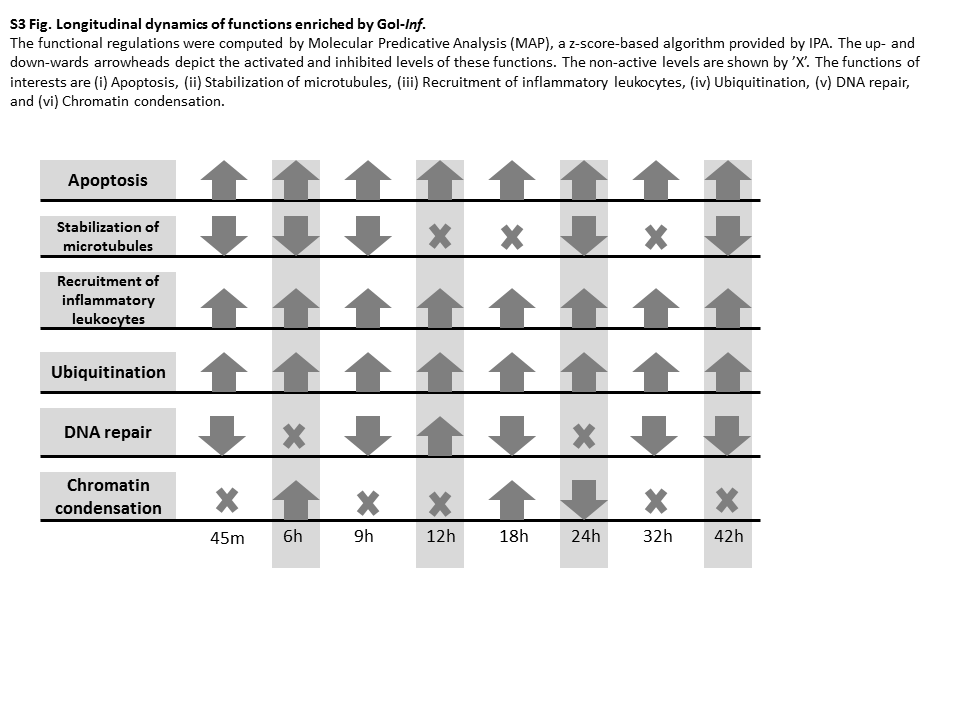

Supplement: S3 Fig — The up- and down-wards arrowheads depict the activated and inhibited levels of these functions. The non-active levels are shown by ‘X’. The functions of interests are (i) Apoptosis, (ii) Stabilization of microtubules, (iii) Recruitment of inflammatory leukocytes, (iv) Ubiquitination, (v) DNA repair, and (vi) Chromatin condensation. (TIF) [file pone.0151788.s003.TIF]

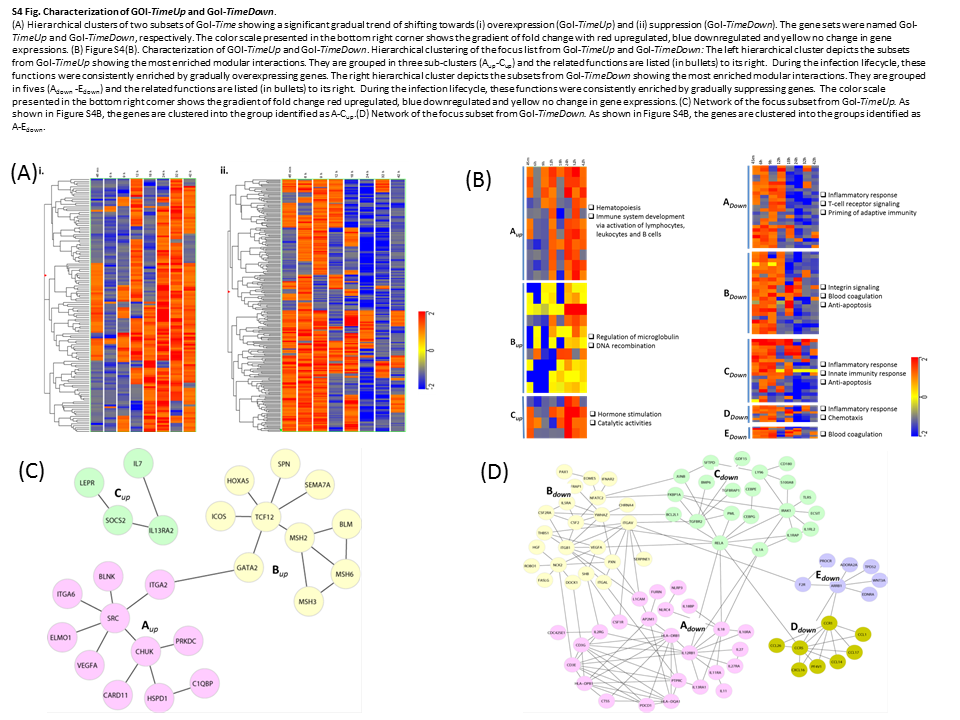

Supplement: S4 Fig — (A) Hierarchical clusters of two subsets of GoI-Time showing a significant gradual trend of shifting towards (i) overexpression (GoI-TimeUp) and (ii) suppression (GoI-TimeDown). The gene sets were named GoI-TimeUp and GoI-TimeDown, respectively. The color scale presented in the bottom right corner shows the gradient of fold change with red upregulated, blue downregulated and yellow no change in gene expressions. (B) Figure S4(B). Characterization of GOI-TimeUp and GoI-TimeDown. Hierarchical clustering of the focus list from GoI-TimeUp and GoI-TimeDown: The left hierarchical cluster depicts the subsets from GoI-TimeUp showing the most enriched modular interactions. They are grouped in three sub-clusters (Aup-Cup) and the related functions are listed (in bullets) to its right. During the infection lifecycle, these functions were consistently enriched by gradually overexpressing genes. The right hierarchical cluster depicts the subsets from GoI-TimeDown showing the most enriched modular interactions. They are grouped in fives (Adown -Edown) and the related functions are listed (in bullets) to its right. During the infection lifecycle, these functions were consistently enriched by gradually suppressing genes. The color scale presented in the bottom right corner shows the gradient of fold change red upregulated, blue downregulated and yellow no change in gene expressions. (C) Network of the focus subset from GoI-TimeUp. As shown in Figure S4B, the genes are clustered into the group identified as A-Cup.(D) Network of the focus subset from GoI-TimeDown. As shown in Figure S4B, the genes are clustered into the groups identified as A-Edown. (TIF) [file pone.0151788.s004.TIF]

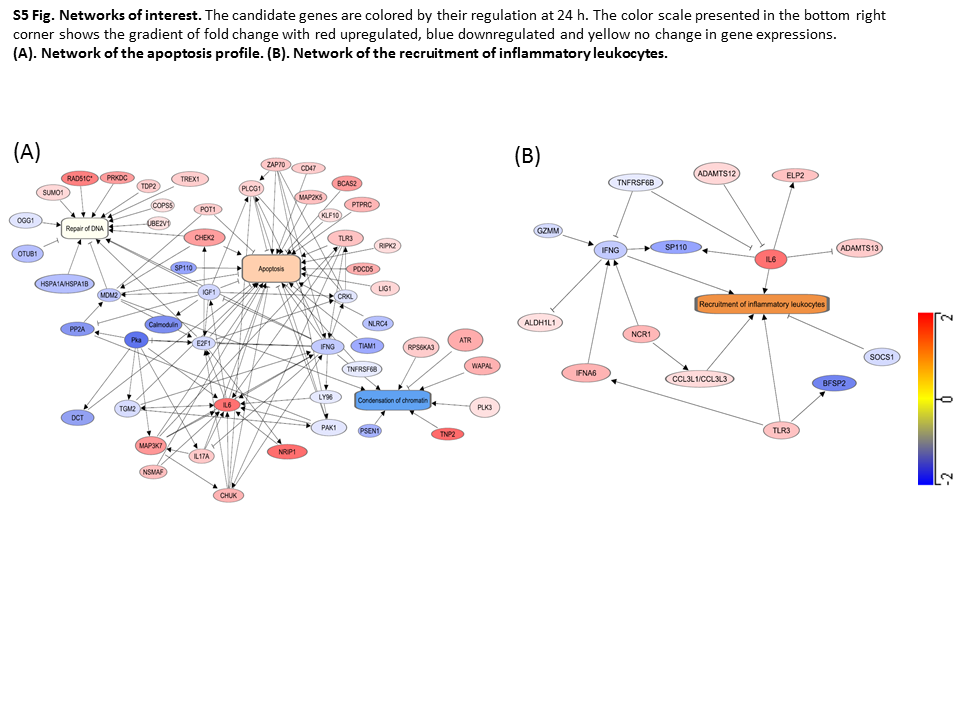

Supplement: S5 Fig — The candidate genes are colored by their regulation at 24 h. The color scale presented in the bottom right corner shows the gradient of fold change with red upregulated, blue downregulated and yellow no change in gene expressions. (A). Network of the apoptosis profile. (B). Network of the recruitment of inflammatory leukocytes. (TIF) [file pone.0151788.s005.TIF]
